# Early diagnostic value of neurotransmitter changes in vulnerable brain regions of patients with subjective cognitive decline detected using proton magnetic resonance spectroscopy

**DOI:** 10.1097/MD.0000000000041474

**Published:** 2025-02-21

**Authors:** Zhen Zeng, Jing He, Tao Yao

**Affiliations:** a Department of Neurology, Wuhan Third Hospital, Wuhan, Hubei, China.

**Keywords:** cognitive impairment, early diagnosis, glutamate, posterior cingulate gyrus, proton magnetic resonance spectroscopy, subjective cognitive decline

## Abstract

This study utilized proton magnetic resonance spectroscopy (1H-MRS) to analyze changes in glutamate-to-creatine ratios (Glu/Cr) in specific brain regions of patients with subjective cognitive decline (SCD) and explored their clinical value for early diagnosis and intervention. Sixty participants, including 30 SCD patients and 30 healthy controls (HCs), were enrolled. Brain imaging was performed using a 3.0T superconducting magnetic resonance scanner. Single-voxel point-resolved spectroscopy sequence (PRESS) was applied for 1H-MRS, focusing on the left posterior cingulate gyrus, hippocampus, and temporal lobe. Glu/Cr ratios were calculated and compared between groups. Correlations between Glu/Cr ratios and cognitive function scores were analyzed. Glu/Cr ratios in the left posterior cingulate gyrus were significantly lower in the SCD group compared to the healthy control group (*P* = .002), suggesting early metabolic disturbances in this region. However, no significant differences in Glu/Cr ratios were observed in the left hippocampus (*P* = .312) or temporal lobe (*P* = .073). Additionally, the Glu/Cr ratio in the posterior cingulate gyrus negatively correlated with cognitive function scores (r = −0.45, *P* < .001), further emphasizing its potential as a biomarker for early cognitive impairment. This study highlights the diagnostic value of reduced Glu/Cr ratios in the posterior cingulate gyrus for identifying SCD patients at risk of cognitive decline. The findings support the application of 1H-MRS as a noninvasive tool for early detection and monitoring of metabolic changes associated with neurodegenerative processes. Future longitudinal studies are needed to confirm these findings and explore their utility in guiding clinical interventions.

## 1. Introduction

With the increasing trend of population aging, cognitive impairment has emerged as a significant global health concern. Subjective cognitive decline (SCD) refers to a self-perceived decline in cognitive abilities, particularly memory, without objective evidence of impairment on standardized cognitive tests.^[1–3]^ SCD is widely recognized as a potential preclinical stage of Alzheimer disease (AD) or other neurodegenerative conditions, highlighting the critical importance of early detection and intervention. Identifying SCD at an early stage offers an opportunity to delay progression to more severe cognitive decline, such as mild cognitive impairment (MCI) or dementia. Early diagnosis also facilitates personalized treatment strategies, ultimately improving patients’ quality of life.^[4–6]^

Proton magnetic resonance spectroscopy (1H-MRS) is a noninvasive imaging technique that quantifies brain metabolites, providing valuable insights into neuronal function and metabolism.^[7–10]^ Among these metabolites, glutamate (Glu), the brain’s primary excitatory neurotransmitter, plays a pivotal role in synaptic transmission, learning, and memory. Dysregulation of Glu levels is associated with synaptic dysfunction and neuronal damage, key pathological features in the early stages of cognitive impairment, making it a promising biomarker for early disease detection.^[11–16]^ Creatine (Cr), a stable reference metabolite, enables normalization of Glu levels, reducing variability across individuals and ensuring reliable metabolic comparisons. By analyzing the Glu-to-Cr ratio (Glu/Cr), metabolic changes in the brain can be quantitatively assessed with greater accuracy.

The hippocampus, posterior cingulate gyrus, and temporal lobe are brain regions particularly vulnerable to early pathological changes in cognitive impairment. The hippocampus is essential for memory formation, while the posterior cingulate gyrus is involved in memory retrieval and default mode network (DMN) activity. The temporal lobe, on the other hand, is critical for processing episodic memory and language. Metabolic disturbances in these regions are considered early indicators of cognitive decline, as seen in SCD. Investigating Glu levels in these regions using 1H-MRS may provide crucial insights into the underlying mechanisms of SCD and offer new opportunities for early diagnosis.

This study aims to analyze changes in Glu levels in vulnerable brain regions of SCD patients compared to a healthy control (HC) group using 1H-MRS technology. By exploring the relationship between Glu levels and cognitive function, we aim to establish new objective biomarkers for the early diagnosis of SCD and a theoretical basis for clinical intervention and treatment evaluation. Furthermore, examining metabolic changes in regions commonly affected by SCD will deepen our understanding of its pathophysiology and provide a foundation for developing novel treatment strategies targeting early neurodegenerative processes.

In summary, this study addresses a critical gap in early diagnostic methods for cognitive impairment by leveraging 1H-MRS to investigate brain metabolite alterations in SCD. It holds significant theoretical and practical value, potentially advancing both research and clinical applications in the prevention and management of SCD.

## 2. Materials and methods

### 2.1. Study participants

This retrospective study was approved by the Ethics Committee of Wuhan Third Hospital. A total of 60 subjects were included in this study, comprising 30 patients with subjective cognitive decline (SCD) as the cognitive impairment group, and 30 HCs as another group. All subjects were from the outpatient and inpatient departments of our hospital, aged between 50 and 80 years, with no gender restrictions.

Cognitive impairment group: 30 SCD patients were included in this group. SCD patients were diagnosed based on the criteria established by the International Working Group (IWG-2), which include: self-reported memory or cognitive decline noticed by the individual over the past months or years, concerns about cognitive decline that are unrelated to external stressors, normal performance on standardized cognitive assessments adjusted for age, gender, and education level, and absence of other conditions that could explain cognitive symptoms, such as depression or neurological disorders.

Healthy control group: Thirty healthy examination patients without cognitive impairment were included. Subjects underwent cognitive function assessment to confirm the absence of memory impairment or other cognitive dysfunction.

There were no significant differences in basic information such as age and gender between the 2 groups, ensuring comparability between the groups.

### 2.2. Inclusion and exclusion criteria

Inclusion criteria: Aged between 50 and 80 years; signed informed consent and voluntary participation in the study; able to complete the 1H-MRS examination and cognitive function assessment.

Exclusion criteria: History of definite neurological diseases such as stroke, Parkinson disease, etc; severe mental illness or psychological disorders; contraindications for magnetic resonance imaging, such as presence of metallic implants in the body, claustrophobia, etc; inability to cooperate with all required examinations for the study.

### 2.3. Measurement indices

This study primarily measured the levels of glutamate (Glu) and creatine (Cr) concentration in the left hippocampus, posterior cingulate gyrus, and temporal lobe. Glutamate, as an important excitatory neurotransmitter, is closely related to cognitive function. Creatine, as a stable metabolite, serves as an internal reference substance for calculating the relative concentration of Glu.

### 2.4. Measurement methods

In this study, all subjects underwent detailed examinations using a 3.0T superconducting magnetic resonance scanner. This advanced equipment provides high-resolution brain images, aiding in a more precise understanding of the subjects’ brain structure and function.

The examination process began with conventional T1- and T2-weighted imaging. T1-weighted imaging highlights the longitudinal relaxation time differences of brain tissues, while T2-weighted imaging mainly reflects differences in transverse relaxation time. The combination of these 2 imaging methods helps observe the anatomical structure of the brain and preliminarily determine the presence of abnormal lesions. Subsequently, we employed fluid-attenuated inversion recovery (FLAIR) sequence scanning. FLAIR technology suppresses the high signal of cerebrospinal fluid, making brain parenchymal lesions more prominent, particularly effective for detecting brain edema, inflammation, etc. Through this step, we could further exclude other potential neurological diseases, ensuring the accuracy and reliability of the study.

After completing the routine scans mentioned above, we performed single-voxel point-resolved spectroscopy (PRESS) for 1H-MRS scanning. 1H-MRS is a noninvasive technique that measures the concentration of different metabolites in the brain. With this technology, we can understand metabolic changes in the brain under different conditions, thus inferring the functional state of the brain. In the 1H-MRS scan, we selected the left hippocampus, posterior cingulate gyrus, and temporal lobe as the regions of interest (VOIs).

During the scanning process, specialized software was used to process the scanning data. Baseline correction was first performed to eliminate the influence of instrument noise and background signals on the results. Subsequently, phase correction was carried out to ensure the accurate position of metabolite peaks. Finally, quantitative analysis of metabolites was performed by calculating the ratio of metabolite peak area to creatine (Cr) peak area to obtain the relative concentration of metabolites. Creatine (Cr), as a stable metabolite, has a relatively stable concentration in the brain and is commonly used as an internal reference substance. By calculating the ratio of other metabolites to Cr, we could eliminate the influence of individual differences and scanning conditions on the results, making the data more comparable between different subjects. Through these steps, we successfully obtained the metabolite concentration data of the bilateral hippocampus and frontal lobe for all subjects. These data provided important basis for the subsequent analysis of differences between SCD patients and HCs.

### 2.5. Statistical methods

Data analysis in this study was performed using SPSS version 30.0 (Chicago) statistical software. Measurement data were expressed as mean ± standard deviation, and the comparison between the cognitive impairment group and the HC group was conducted using independent sample *t*-tests. Pearson correlation analysis was used for correlation analysis. A significance level of *α* = 0.05 was set for all statistical analyses.

## 3. Results

### 3.1. Baseline characteristics comparison

As shown in Table 1, there were no significant differences in baseline characteristics between the subjective cognitive decline (SCD) group (n = 30) and the HC group (n = 30) (*P* > .05), ensuring comparability between the groups. The mean age of the SCD and control groups was 65.2 ± 7.3 years and 64.8 ± 7.1 years, respectively (*t* = 0.20, *P* = .84); the gender distribution (male/female) was 14/16 and 15/15 (χ²=0.07, *P* = .79); and the years of education were 10.5 ± 3.2 and 10.8 ± 3.1 (*t* = 0.31, *P* = .76). Furthermore, there were no significant differences in body mass index (BMI), prevalence of hypertension, diabetes, smoking history, mini-mental state examination scores, or depression scores (CES-D) between the 2 groups, with *P*-values all >.05. These results confirm that the groups were balanced in demographic, health, and cognitive characteristics, minimizing the risk of confounding factors affecting the study outcomes.

**Table 1:** Baseline characteristics of the study population.

Variable	Cognitive impairment group (n = 30)	HC group (n = 30)	Statistical value	*P*-value
Age (yrs)	65.2 ± 7.3	64.8 ± 7.1	*t* = 0.20	.84
Gender (male/female)	14/16	15/15	χ² = 0.07	.79
Education (yrs)	10.5 ± 3.2	10.8 ± 3.1	*t* = 0.31	.76
BMI, kg/m²	23.8 ± 2.5	23.5 ± 2.7	*t* = 0.42	.68
Hypertension (%)	10 (33.3%)	9 (30.0%)	χ² = 0.08	.78
Diabetes (%)	4 (13.3%)	3 (10.0%)	χ² = 0.16	.68
Smoking history (%)	7 (23.3%)	6 (20.0%)	χ² = 0.09	.77
MoCA	28.5 ± 1.2	28.8 ± 1.1	*t* = 0.59	.56
Depression (CES-D score)	8.1 ± 3.2	7.8 ± 2.9	*t* = 0.33	.74

BMI = body mass index, HC = healthy control, MoCA = Montreal cognitive assessment.

### 3.2. Comparison of metabolite concentrations

Table 2 summarizes the comparison of Glu/Cr ratios in the left posterior cingulate gyrus, left hippocampus, and middle temporal gyrus between the subjective cognitive decline (SCD) group and the HC group. The results indicate that the Glu/Cr ratio in the left posterior cingulate gyrus of the SCD group (0.40 ± 0.09) was significantly lower than that of the HC group (0.46 ± 0.05), with a statistically significant difference (*t* = −3.19, *P* = .002). This suggests that reduced Glu/Cr ratios in the posterior cingulate gyrus may reflect early metabolic disturbances associated with cognitive impairment.

**Table 2:** Comparison of metabolite concentrations between cognitive impairment group and healthy control group.

Group	N	Left cingulate retrosplenial cortex	Left hippocampus	Middle temporal gyrus
Cognitive impairment group	30	0.40 ± 0.09	0.44 ± 0.09	0.41 ± 0.09
Healthy control group	30	0.46 ± 0.05	0.45 ± 0.05	0.42 ± 0.05
*t*		4.32	0.815	0.614
*P*		.002	.312	.073

In contrast, no significant differences were observed in the Glu/Cr ratios between the 2 groups in the left hippocampus (*t* = 0.815, *P* = .312) or the middle temporal gyrus (*t* = 0.614, *P* = .073). These findings imply that the metabolic changes in the left posterior cingulate gyrus are more pronounced and potentially more relevant to the early stages of cognitive decline compared to other regions.

### 3.3. Correlation between metabolite concentrations and cognitive function

Pearson correlation analysis was performed to investigate the correlation between metabolite concentrations in different brain regions and cognitive function scores in the cognitive impairment group and the HC group. In the left posterior cingulate gyrus region, the Glu/Cr ratio was negatively correlated with cognitive function scores (*r* = −0.45, *P* < .001), indicating that lower metabolite concentrations in this region may be associated with poorer cognitive performance. However, in the left hippocampus and temporal lobe regions, the correlation between Glu/Cr ratio and cognitive function scores was not significant in either the cognitive impairment group or the HC group (*P*-values were .13 and .24, respectively). This may be due to the complex relationship between metabolite concentrations and cognitive function in these regions or the influence of other factors (Table 3).

**Table 3:** Correlation between metabolite concentrations and cognitive function scores in different groups.

Region	Metabolite	Cognitive impairment group vs HC group	Correlation coefficient (*r*)	*P*
Left cingulate retrosplenial cortex	Glu/Cr	35 vs 85	−0.45	<.001
Left hippocampus	Glu/Cr	35 vs 85	0.72	.13
Middle temporal gyrus	Glu/Cr	35 vs 85	0.53	.24

HC = healthy control.

### 3.4. Comparison of metabolite concentrations between cognitive impairment patients and healthy controls

Table 4 further compares the concentrations of Glu and Cr, 2 types of metabolites, in the left posterior cingulate gyrus, left hippocampus, and temporal lobe between the cognitive impairment group and the healthy control group. In the left posterior cingulate gyrus region, the Glu concentration of the cognitive impairment group (1.25 ± 0.16) was significantly lower than that of the HC group (1.56 ± 0.15), with a *t*-value of 4.76 and a *P*-value <.001, indicating a significant statistical difference. However, there was no significant difference in Cr concentration between the 2 groups (*P* = .57). In the left hippocampus and temporal lobe regions, there were no significant differences in either Glu or Cr concentrations between the 2 groups (*P*-values were all >.05). These results further support the findings in Tables 2 and 3, indicating that the abnormal concentration of metabolites in the left posterior cingulate gyrus region may be related to cognitive decline.

**Table 4:** Comparison of metabolite concentrations between cognitive impairment group and HC group.

Metabolite	Region	Cognitive impairment group	HC group	*t*-value	*P*
Glu	Left cingulate retrosplenial cortex	1.25 ± 0.16	1.56 ± 0.15	4.76	<.001
Cr	Left cingulate retrosplenial cortex	3.13 ± 0.29	3.39 ± 0.28	0.512	.57
Glu	Left hippocampus	1.67 ± 0.06	1.69 ± 0.01	0.316	.41
Cr	Left hippocampus	3.80 ± 0.31	3.76 ± 0.32	0.613	.62
Glu	Middle temporal gyrus	1.55 ± 0.09	1.61 ± 0.05	0.473	.43
Cr	Middle temporal gyrus	3.78 ± 0.27	3.83 ± 0.26	0.637	.49

HC = healthy control.

## 4. Discussion

This study examined changes in Glu/Cr ratios in the left posterior cingulate gyrus, hippocampus, and temporal lobe of patients with subjective cognitive decline (SCD) using 1H-MRS. As a potential preclinical stage of AD, SCD is characterized by subtle cognitive changes that may precede structural brain alterations. Identifying early metabolic disruptions, such as changes in Glu/Cr ratios, is critical for understanding the pathophysiology of SCD and developing biomarkers for early detection. These findings provide valuable insights into region-specific metabolic alterations and their implications for early diagnosis and targeted intervention.

### 4.1. Metabolic changes in the posterior cingulate gyrus

Our results indicate a significant reduction in the Glu/Cr ratio in the left posterior cingulate gyrus of the SCD group compared to the healthy control (HC) group (*P* = .002). Glutamate, as the brain’s primary excitatory neurotransmitter, plays a pivotal role in synaptic plasticity, memory, and learning.^[17–19]^ Its reduction, reflected by a lower Glu/Cr ratio, may indicate neuronal dysfunction and impaired synaptic transmission. This metabolic disturbance aligns with prior findings that posterior cingulate gyrus dysfunction is an early marker of cognitive impairment, particularly in preclinical AD.^[20–22]^ Additionally, the significant reduction in absolute Glu concentrations (*P* < .001) in this region further supports its vulnerability in SCD patients, emphasizing its diagnostic value.

### 4.2. Regional specificity of metabolic alterations

Unlike the posterior cingulate gyrus, the left hippocampus and temporal lobe showed no significant differences in Glu/Cr ratios or absolute metabolite concentrations between the SCD and HC groups. This discrepancy highlights the unique vulnerability of the posterior cingulate gyrus, which is a critical hub within the DMN. The DMN is highly sensitive to early neurodegenerative changes, such as amyloid deposition and synaptic dysfunction, which are characteristic of preclinical AD. In contrast, metabolic changes in the hippocampus and temporal lobe may emerge later in the disease process or require more advanced stages of cognitive impairment to manifest. These findings align with prior studies showing that posterior cingulate gyrus dysfunction often precedes structural or metabolic changes in other regions.^[22–26]^ This regional specificity highlights the posterior cingulate gyrus as a more sensitive marker for detecting early cognitive decline in SCD patients.

### 4.3. Correlation between Glu/Cr ratios and cognitive function

The negative correlation between the Glu/Cr ratio in the posterior cingulate gyrus and cognitive function scores (r = −0.45, *P* < .001) underscores its potential as a biomarker for cognitive performance. This finding is consistent with previous research demonstrating that reduced glutamate levels are associated with diminished cognitive abilities, likely due to impaired synaptic function and network connectivity in the DMN, where the posterior cingulate gyrus plays a central role.^[27–29]^ In contrast, the lack of significant correlations in the hippocampus and temporal lobe may reflect the complex interplay between these regions and cognitive processes, as well as compensatory mechanisms that mask early metabolic changes.^[30]^

### 4.4. Clinical implications and future directions

This study underscores the importance of 1H-MRS as a noninvasive tool for detecting early metabolic changes in SCD. The significant reduction in Glu/Cr ratios in the posterior cingulate gyrus highlights its potential as a biomarker for identifying individuals at risk of cognitive decline. First, this biomarker could facilitate early diagnosis of SCD, enabling timely interventions to slow disease progression and improve patient outcomes. Second, Glu/Cr ratios may serve as a valuable tool for monitoring the efficacy of therapeutic interventions, such as cognitive training or pharmacological treatments targeting early neurodegenerative changes. Finally, integrating this biomarker into routine cognitive screening protocols for individuals with subjective memory complaints could optimize early detection and stratification, thereby improving the allocation of healthcare resources. However, the cross-sectional nature of this study limits causal inferences, and longitudinal studies are needed to confirm whether these metabolic changes predict cognitive trajectories or progression to more severe conditions, such as MCI or AD.^[31]^

Additionally, exploring the interplay between metabolic disturbances and other factors, such as neuroinflammation, vascular contributions, and amyloid pathology, could provide a more comprehensive understanding of the mechanisms underlying SCD. Future research should also investigate additional brain regions and employ multimodal imaging approaches to validate and expand upon these findings.^[28,32]^

There are some limitations to this study that need to be noted. First, although we controlled for key confounders as much as possible by including criteria and matching between groups, such as ensuring that the SCD group did not differ significantly from the HC group in variables such as age, sex, education level, and BMI, and excluding patients with obvious neurological or psychiatric disorders that could affect cognition and brain metabolism, However, certain factors that are not observed or are difficult to fully control (such as mood swings or individual lifestyle differences) may still have a slight influence on the results. Second, due to the cross-sectional design of the study, it is not possible to establish a causal relationship between metabolic changes and cognitive decline. In the future, longitudinal studies can be combined with a larger sample size and the introduction of multivariate regression models to further verify the predictive ability of changes in metabolite levels on cognitive function, and to comprehensively control potential confounding factors, so as to enhance the robustness and generalization of research conclusions.

## 5. Conclusion

In conclusion, this study reveals that reduced Glu/Cr ratios in the posterior cingulate gyrus are a prominent metabolic alteration in SCD patients and may serve as a valuable biomarker for early cognitive impairment. These findings contribute to a growing body of evidence supporting the use of 1H-MRS in the early detection of neurodegenerative processes and provide a foundation for future studies to advance the prevention and management of cognitive decline.

## Author contributions

**Conceptualization:** Zhen Zeng, Jing He, Tao Yao.

**Data curation:** Zhen Zeng, Jing He, Tao Yao.

**Formal analysis:** Zhen Zeng, Jing He, Tao Yao.

**Investigation:** Jing He, Tao Yao.

**Methodology:** Jing He, Tao Yao.

**Writing – original draft:** Tao Yao.

**Writing – review & editing:** Tao Yao.
